# Anatomical landmarks for ankle block

**DOI:** 10.1186/s13018-023-04039-2

**Published:** 2023-09-07

**Authors:** K. V. H. Nimana, A. M. D. S. R. U. Senevirathne, R. Pirannavan, M. P. S. Fernando, U. A. Liyanage, K. A. Salvin, A. P. Malalasekera, Y. Mathangasinghe, D. J. Anthony

**Affiliations:** 1 Department of Anatomy, Genetics and Biomedical Informatics, Faculty of Medicine, University of Colombo, Colombo, Sri Lanka; 2 Department of Anatomy, Faculty of Medicine, University of Kelaniya, Kelaniya, Sri Lanka

**Keywords:** Anatomy, Lower extremity, Regional anaesthesia

## Abstract

We aimed to describe anatomical landmarks to accurately locate the five nerves that are infiltrated to accomplish anaesthesia of the foot in an ankle block. Twenty-four formaldehyde-fixed cadaveric ankles were studied. Photographs of cross sections of the frozen legs, cut at a horizontal plane across the most prominent points of the medial and lateral malleoli, were analysed. The curvilinear distance from the most prominent point of the closest malleolus to each of the five cutaneous nerves and their depth from the skin surface were measured. Sural, tibial, deep peroneal, saphenous and medial dorsal cutaneous nerves were located 5.2 ± 1.3, 9.2 ± 2.4, 7.4 ± 1.9, 2.8 ± 1.1, 2.1 ± 0.6 mm deep to the skin surface. The curvilinear distances from the medial malleolus to the tibial, deep peroneal and saphenous nerves were 32.5 ± 8.9, 62.8 ± 11.1 and 24.4 ± 7.9 mm, respectively. The curvilinear distances from the lateral malleolus to the sural and medial dorsal cutaneous branches of superficial peroneal nerves were 27.9 ± 6.3 and 52.7 ± 7.3 mm, respectively. The deep peroneal nerve was found between the tendons of the extensor hallucis longus and the extensor digitorum longus in the majority of specimens, while the medial dorsal cutaneous nerve was almost exclusively found on the extensor digitorum longus tendon. The sural and tibial nerves were located around halfway between the most prominent point of the relevant malleolus and the posterior border of the Achilles tendon. In conclusion, this study describes easily identifiable, palpable bony and soft tissue landmarks that could be used to locate the nerves around the ankle.

**Supplementary Information:**

The online version contains supplementary material available at 10.1186/s13018-023-04039-2.

## Main text

Regional anaesthesia for foot surgeries can be achieved by blocking the five cutaneous nerves of the foot at the ankle level, a procedure known as "ankle block". Similar to general anaesthesia, ankle block helps achieve high-quality pain relief in foot surgeries while reducing the need for opioids and increasing patient satisfaction [1, 2]. Moreover, with the increasing prevalence of the aged population and growing incidence of obesity and diabetes-related morbidity, the use of regional anaesthesia for foot surgeries is likely to expand in the future [3].

Ultrasound guidance has enabled precise and selective delivery of ankle blocks over the last few decades. However, the success rates of image-based techniques compared to conventional landmark-based techniques for the ankle block are controversial [4]. Moreover, the landmark-based technique is still widely practised in resource-poor settings [5]. Therefore, the explicit knowledge of anatomical landmarks to locate these nerves may help reduce the risk of infiltrating regional vessels, thus minimizing the side effects of anaesthetics. Here, we report the anatomical landmarks to accurately locate the nerves for ankle blocks.

## Methods

**Fig. 1:**
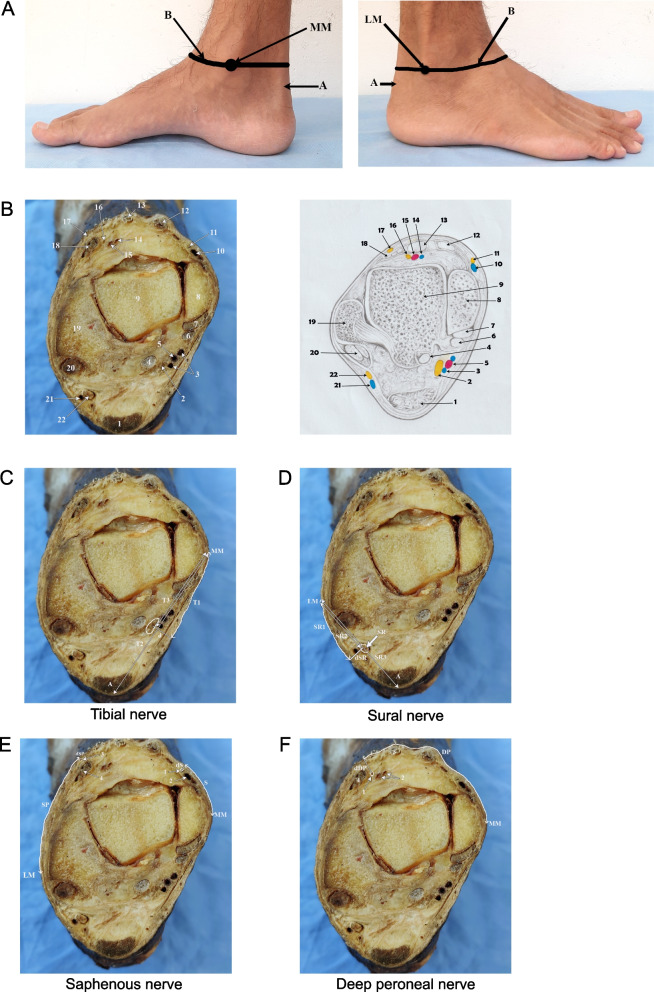
Anatomical landmarks for ankle block. **A** Medial and lateral views of the foot and leg. B, the horizontal line drawn across the *most prominent point* of the medial malleolus (MM) and the *most prominent point* of the lateral malleolus (LM), which is parallel to the knee joint line; A, Achilles tendon. **B** Cross section of the ankle at a horizontal plane across the *most prominent point* of the medial malleolus and the *most prominent point* of the lateral malleolus, which is parallel to the knee joint line. 1, Achilles tendon; 2, tibial nerve; 3, posterior tibial veins; 4, flexor hallucis longus; 5, posterior tibial artery; 6, flexor digitorum longus; 7, tibialis posterior; 8, medial malleolus; 9, talus; 10, great saphenous vein; 11, saphenous nerve; 12, tibialis anterior; 13, extensor hallucis longus; 14, anterior tibial vein; 15, anterior tibial artery; 16, deep peroneal nerve; 17, medial dorsal cutaneous nerve; 18, extensor digitorum longus; 19, lateral malleolus; 20, peroneus longus and brevis; 21, small saphenous vein; 22, sural nerve. **C** Cross section of the ankle across the plane described in methods, showing the measurements taken for the tibial nerve. MM, the most prominent point of the medial malleolus; T1, the curvilinear distance from the MM to the point where the perpendicular line drawn from the middle of the tibial nerve meets the skin surface; T2, the direct distance from the MM to the posterior border of the Achilles tendon; T3, the direct distance from the MM to the point where the perpendicular line drawn from the middle of the tibial nerve meets the T2 line; dT, the perpendicular distance from the tibial nerve to the skin surface; A, Achilles tendon. **D** As in C, measurements to the sural nerve. LM, the most prominent point of the lateral malleolus; SR1, the curvilinear distance from the LM to the point where the perpendicular line drawn from the middle of the sural nerve meets the skin surface; SR2, the direct distance between the LM and the posterior border of the Achilles tendon; SR3, the direct distance from the LM to the point where the perpendicular line drawn from the middle of the sural nerve meets the SR2 line; dSR, the perpendicular distance from the sural nerve to the skin surface; SR, sural nerve; A, Achilles tendon. **E** As in C**,** measurements to the saphenous nerve and medial dorsal cutaneous nerve. MM, most prominent point of the medial malleolus; LM, most prominent point of the lateral malleolus; S, the curvilinear distance from the MM to the point where the perpendicular line drawn from the middle of the saphenous nerve meets the skin surface; dS, perpendicular distance from the saphenous nerve to the skin surface; SP, curvilinear distance from the LM to the point where the perpendicular line drawn from the middle of the medial dorsal cutaneous nerve meets the skin surface; dSP, the perpendicular distance from the medial dorsal cutaneous nerve to the skin surface; 1, saphenous nerve; 2, great saphenous vein.; 3, medial dorsal cutaneous nerve; 4, extensor digitorum longus tendon. **F** As in C, measurements to the deep peroneal nerve. MM, the most prominent point of the medial malleolus; DP, the curvilinear distance measured from MM to the point where the perpendicular line drawn from the middle of the nerve meets the skin surface; dDP, the perpendicular distance from the deep peroneal nerve to the skin surface; 1, deep peroneal nerve; 2, anterior tibial artery and vein; 3, extensor hallucis longus tendon; 4, extensor digitorum longus tendon

A descriptive cross-sectional study was conducted on self-donated cadavers of Sri Lankan ethnicity. The study was approved by the institutional Ethics Review Committee [EC/21/100]. The sample size was calculated according to Lwanga and Lemeshow [6] with a 95% confidence level and a 5% margin of error using population estimates of a previous study [7]. The required minimum sample size was 22. We randomly selected 24 lower limbs from independent cadavers, which have been preserved as described elsewhere [8]. Lower limbs with deformities, a history of fractures and previous surgeries were excluded. After obtaining the mid-calf circumference using a measuring tape, the cadavers were frozen for 24 h at − 20°C. Subsequently, they were sectioned using an electric saw at a horizontal plane across the most prominent point of the medial malleolus (hereafter referred to as “MM”) and the most prominent point of the lateral malleolus (hereafter referred to as “LM”), which is parallel to the knee joint line (Fig. 1A). After identifying the nerves, the specimens were stabilized, and photographs were obtained using a digital camera from a fixed distance (Fig. 1B). The measurements from the anatomical landmarks to the respective nerves were obtained from photographs of the cross sections using Fiji (v1.53) (see Additional file 1: Methods). The measurements were presented as mean ± standard deviation.

## Results

Of the twenty-four ankles, ten were left-sided and fourteen were right-sided. The summary measurements are presented in Additional file 1: Table S1. The mean direct distance from the MM to the posterior border of the Achilles tendon (T2) was 57.9 ± 13.5 mm and the mean direct distance from the MM to the point where the perpendicular line drawn from the middle of the tibial nerve meets the T2 line (T3) was 27.1 ± 7.7 mm. Also, the mean direct distance between the LM and the posterior border of the Achilles tendon (SR2) was 46.4 ± 8.9 mm and the mean direct distance from the LM to the point where the perpendicular line drawn from the middle of the sural nerve meets the SR2 line (SR3) was 24.0 ± 5.5 mm.

The mean curvilinear distance between MM and LM was 126.2 ± 13.5 mm. In a majority of specimens, the deep peroneal nerve (89%) was located lateral to the tendon of extensor hallucis longus (EHL), whereas the medial dorsal cutaneous nerve (90%) was located on the tendon of extensor digitorum longus (EDL). In seventeen out of 24 specimens (70.8%), the tibial nerve was observed posterolateral to the posterior tibial artery with a mean direct distance of 5.3 ± 1.9 mm between them. Though in the majority of the specimens (*n* = 12, 50%), the sural nerve was seen anteromedial to the small saphenous vein, it was also found anterior (*n* = 3, 12.5%), anterolateral (*n* = 3, 12.5%) and posteromedial (*n* = 4, 16.9%) to the vein. However, the small saphenous vein was absent in two specimens (8.3%). The saphenous nerve was located anterolateral to the great saphenous vein in the majority of specimens (*n* = 17, 70.8%), but in only four specimens (16.7%), the nerve was found posteromedial to the vein. However, the great saphenous vein was absent in three specimens (12.5%). Furthermore, the deep peroneal nerve was found anterolateral to the anterior tibial artery in the majority of specimens (*n* = 10, 41.7%), while it was located anteromedial (*n* = 7, 29.2%), posterolateral (*n* = 3, 12.5%) and lateral (*n* = 2, 8.3%) to the artery. The anterior tibial artery was not found in two specimens (8.3%). Only the curvilinear distance from the LM to the point where the perpendicular line drawn from the middle of the sural nerve meets the skin surface (SR1) showed a significant association with the calf circumference (r = 0.6, p = 0.002).

## Discussion

Here, we report anatomical landmarks to locate the five cutaneous nerves of the foot at the level of the ankle joint, which could help deliver ankle blocks precisely. Previous studies have reported the direct distances between the nerves and the bony landmarks at the ankle on dissected cadavers measured by a calliper [7, 9, 10]. In most cases, the investigators have removed the skin completely, and in a few, they have taken measurements from an incision made over the nerve. However, the measurements obtained in a superficially dissected field cannot be accurately inferred from the actual measurements on an intact body surface. Furthermore, the lack of knowledge regarding the depth of the nerve from the skin surface hinders the spatial localization of the nerves. Therefore, in this study, we described the depth of the nerves from the skin surface and the curvilinear distances to the nerves from the bony landmarks in a horizontal plane across the most prominent points of the medial and lateral malleoli. This line is easy to draw even in a supine position just by elevating the leg, without the need to turn the patient. Also, using this plane gives an additional advantage of ensuring a complete block of tibial, sural, and deep peroneal nerves, as typically they divide distal to this level [11, 12].

### Tibial nerve

The tibial nerve is typically blocked by palpating the posterior tibial artery and the needle is slowly advanced posterior to the artery, perpendicular to the skin for 5–20 mm, piercing the flexor retinaculum [13, 14]. We found that in most cases, the tibial nerve is located posterolateral to the posterior tibial artery and 8–10 mm perpendicularly deep to the skin (95% CI). However, there are occasions where clinicians cannot palpate the posterior tibial pulsations as it is one of the most difficult pulses to detect [15]. In such a situation, Schurman [16] proposed injecting one fingerbreadth posterior to the MM, while Sarrafian [10] suggested injecting 10–15 mm anterior to the Achilles tendon to block the tibial nerve. Our study found that the tibial nerve is located 29–36 mm posterior to the most prominent point of the MM. This precise position may help locate the needle entry site, particularly if palpation of the posterior tibial artery is difficult. Schabort [7] reported that the tibial nerve is located approximately at the midpoint between the posterior border of the Achilles tendon and the most medial part of the MM. This finding is comparable to our study as we found the tibial nerve courses approximately halfway between the most prominent part of the MM and the posterior border of the Achilles tendon.

### Deep peroneal nerve

The deep peroneal nerve is traditionally blocked by inserting a needle in a perpendicular direction between the tendons of the tibialis anterior and the EHL at a level immediately above the MM, lateral to the dorsalis pedis artery [10, 13]. However, some suggest inserting the needle immediately lateral to the EHL tendon at the level of the MM [17]. The results of our study also support the existing guidelines, as in most cases, the deep peroneal nerve was found in the interval between the EHL and the EDL tendons. In line, Solomon [18] reported that the nerve is situated between the tendons of EHL and EDL in 40 cases (58.8%) and deep to the extensor digitorum longus in 26 cases (38.2%). This finding is further supported by the observations by Lawrence [11] in which the nerve crosses deep into the EHL tendon at an average distance of about 12.5 mm proximal to the ankle joint to reach the interval between the tendons of EHL and the EDL.

Schabort [7] reported that the deep peroneal nerve was found 32 mm and 37.5 mm lateral to the most anterior aspect of the MM in females and males, respectively. In contrast, we found that the nerve is situated 58–68 mm lateral to the most prominent part of the MM and 6–8 mm perpendicularly deep to the skin. This discrepancy can be attributed to the more medial bony point we used to take measurements and distance being taken along the skin surface rather the direct distance taken after dissection by Schabort [7]. Solomon reported that the deep peroneal nerve was located at the lateral margin of the vascular bundle in the majority of cases (55.9%) while the nerve crossed the ankle joint over the vascular bundle in 26.5% of cases [18]. Similarly, our study found that in the majority of cases (54.2%) the nerve was situated lateral to the anterior tibial artery and medial to the artery in 29.2% of cases. Furthermore, we found that the deep peroneal nerve was located halfway between the most prominent points of the medial and lateral malleoli. This easy-to-remember landmark may help in locating the nerve, particularly in obese individuals where tendons of EHL and EDL cannot be palpable.

### Superficial peroneal nerve

The superficial peroneal nerve divides into the intermediate and medial dorsal cutaneous nerves at the distal one-third of the leg. The previous literature shows that the position of the intermediate dorsal cutaneous nerve (IDCN) at the intermalleolar level is more constant as it runs adjacent to the LM. Therefore, it is blocked by a subcutaneous wheal raised just medial to the LM [12, 19]. However, the position of the medial dorsal cutaneous nerve (MDCN) at the intermalleolar level varies according to the pattern of division. Cousins [20] suggested raising a wheal from the anterior border of the tibia to the superior aspect of the LM, while Beskin [21] suggested introducing the needle two fingerbreadths superior to the tip of the LM and injecting subcutaneously across the anterior border of the fibula and tibia to block this nerve.

In our study, the MDCN was found on the tendon of EDL in almost all specimens and an average curvilinear distance of 68–79 mm from the most prominent point of the MM, 1.8–2.5 mm perpendicularly deep to the skin surface. Uceler [12], who studied the position of the branches of superficial peroneal nerve at the same plane as in our study, found MDCN 52.68 ± 6.67 mm lateral to the most prominent point of the MM. Nonetheless, Uceler [12] have reported the direct measurements in contrast to the curvilinear measurements in our study which could have resulted in this discrepancy. However, in terms of the distance between the malleoli, the findings of their study support our observation that MDCN lies at 58% of the intermalleolar curvilinear distance from the MM.

### Sural nerve

Ferrera [22] suggested that the sural nerve blockade can be achieved by infiltrating the local anaesthetics subcutaneously just anterior to the Achilles tendon at the LM level. Schabort [7] reported that the sural nerve is found approximately 25 mm and 20 mm posterior to the inferior tip of the LM in males and females, respectively. Conversely, we found that the sural nerve lies 25–30 mm posterior to the most prominent point of the LM along the skin surface and 4–6 mm perpendicularly deep to the skin. Furthermore, in line with the suggestion of Hromadka [9], we report that the sural nerve is located approximately midway between the most prominent point of the LM and the posterior border of the Achilles tendon.

### Saphenous nerve

Current guidelines advise infiltrating local anaesthetics approximately 2 cm anterior and posterior to the great saphenous vein immediately above the MM to block the saphenous nerve [17]. When the great saphenous vein is not visible or palpable, Hoerster recommended forming a subcutaneous ring block from the edge of the tibia to the Achilles tendon about a hand’s breadth above the MM to block the saphenous nerve [14]. In a South African population, the saphenous nerve was found to be about 1 cm (11.5 mm for males and 10 mm for females) from the most anterior aspect of the MM [10]. According to a study in West Indies [23], the terminal branch of the saphenous nerve was observed 0.25–0.65 cm anterior to the midpoint of the anterior border of the MM. In contrast, we found that the saphenous nerve lies 2–2.8 cm anterior to the most prominent point of the MM. The discrepancy observed here can be attributed to the different bony points and curvilinear distance we measured in our study.

In conclusion, this study describes easily identifiable, palpable bony and soft tissue landmarks that could be used to locate the nerves around the ankle, which could help deliver safe ankle blocks. The tibial nerve can be blocked by infiltrating local anaesthetic 8–10 mm perpendicularly deep into the skin, either just posterior to the posterior tibial pulse behind the MM or inserting the needle in the midway between the most medial point of the MM and the posterior border of the Achilles tendon in a horizontal plane when the pulse is not palpable. The deep peroneal nerve can be precisely blocked by infiltrating at the interval between the tendons of the EHL and the EDL at the intermalleolar level, 6–8 mm perpendicularly deep to the skin. The MDCN can be blocked by making a subcutaneous wheal of about 1 cm over the EDL tendon. The sural nerve can be blocked by infiltrating local anaesthetics halfway between the most prominent point of the LM and the posterior border of the Achilles tendon, 4–6 mm perpendicularly deep to the skin. The saphenous nerve can be blocked by making a subcutaneous wheal of about 1 cm, a thumb breadth anterior to the most prominent point of the MM.

### Supplementary Information

**Additional file 1.** **Table S1**: The curvilinear distances are provided in relation to the most prominent parts of the medial and lateral malleoli. The depth is the distance between the nerve and the overlying skin (See Figure 1 for the definitions). All the measurements were obtained in millimetres. Abbreviations: T1, curvilinear distance from the MM to the point where the perpendicular line drawn from the middle of the tibial nerve meets the skin surface; DP, curvilinear distance measured from MM to the point where the perpendicular line drawn from the middle of the deep peroneal nerve meets the skin surface; S, curvilinear distance from the MM to the point where the perpendicular line drawn from the middle of the saphenous nerve meets the skin surface; SR1, curvilinear distance from the LM to the point where the perpendicular line drawn from the middle of the sural nerve meets the skin surface; SP, curvilinear distance from the LM to the point where the perpendicular line drawn from the middle of the medial dorsal cutaneous nerve meets the skin surface; dT, perpendicular distance from the tibial nerve to the skin surface; dDP, perpendicular distance from the deep peroneal nerve to the skin surface; dS, perpendicular distance from the saphenous nerve to the skin surface; dSR, perpendicular distance from the sural nerve to the skin surface; dSP, perpendicular distance from the medial dorsal cutaneous nerve to the skin surface.

## Data Availability

The datasets used and/or analysed during the current study are available from the corresponding author upon reasonable request.
