# Supplementary material for: Anatomical landmarks for ankle block
Source: J Orthop Surg Res. 2023 Sep 7;18:665. doi: 10.1186/s13018-023-04039-2 (PMC10483789; doi:10.1186/s13018-023-04039-2)
Supplement: Supplementary file 1 — Additional file 1. Table S1: The curvilinear distances are provided in relation to the most prominent parts of the medial and lateral malleoli. The depth is the distance between the nerve and the overlying skin (See Figure 1 for the definitions). All the measurements were obtained in millimetres. Abbreviations: T1, curvilinear distance from the MM to the point where the perpendicular line drawn from the middle of the tibial nerve meets the skin surface; DP, curvilinear distance measured from MM to the point where the perpendicular line drawn from the middle of the deep peroneal nerve meets the skin surface; S, curvilinear distance from the MM to the point where the perpendicular line drawn from the middle of the saphenous nerve meets the skin surface; SR1, curvilinear distance from the LM to the point where the perpendicular line drawn from the middle of the sural nerve meets the skin surface; SP, curvilinear distance from the LM to the point where the perpendicular line drawn from the middle of the medial dorsal cutaneous nerve meets the skin surface; dT, perpendicular distance from the tibial nerve to the skin surface; dDP, perpendicular distance from the deep peroneal nerve to the skin surface; dS, perpendicular distance from the saphenous nerve to the skin surface; dSR, perpendicular distance from the sural nerve to the skin surface; dSP, perpendicular distance from the medial dorsal cutaneous nerve to the skin surface. [file 13018_2023_4039_MOESM1_ESM.docx]

**SUPPLEMENTARY MATERIALS**

**Supplementary Methods**

A perpendicular line was drawn from the middle of each nerve to the overlying nearest skin surface. The depth of each nerve "d" was defined as the distance between the nerve and the overlying skin, measured along this line. For the tibial nerve, three measurements were taken (Figure 1B). The curvilinear distance from the MM to the point where the perpendicular line drawn from the middle of the tibial nerve meets the skin surface (T1), the direct distance from the MM to the posterior border of the Achilles tendon (T2), and the direct distance from the MM to the point where the perpendicular line drawn from the middle of the tibial nerve meets the T2 line (T3). Three measurements were similarly obtained for the sural nerve (Figure 1B). The curvilinear distance from the LM to the point where the perpendicular line drawn from the middle of the sural nerve meets the skin surface (SR1), the direct distance between the LM and the posterior border of the Achilles tendon (SR2), and the direct distance from the LM to the point where the perpendicular line drawn from the middle of the sural nerve meets the SR2 line (SR3). The saphenous nerve was measured by taking the curvilinear distance from the MM to the point where the perpendicular line drawn from the middle of the nerve meets the skin surface (S) (Figure 1B). For the deep peroneal nerve, the curvilinear distance was measured from MM to the point where the perpendicular line drawn from the middle of the nerve meets the skin surface (DP) (Figure 1B). The medial cutaneous branch of the superficial peroneal nerve was measured by taking the curvilinear distance from the LM to the point where the perpendicular line drawn from the middle of the nerve meets the skin surface (SP) (Figure 1B). In addition, we attempted to explain the relationship between the aforementioned nerves and neighbouring vessels by measuring the direct distance between them as well as the nerve's spatial position relative to the vessel.

**Supplementary Tables**

**Table S1:** The curvilinear distances are provided in relation to the most prominent parts of the medial and lateral malleoli. The depth is the distance between the nerve and the overlying skin (See Figure 1 for the definitions). All the measurements were obtained in millimetres. Abbreviations: T1, curvilinear distance from the MM to the point where the perpendicular line drawn from the middle of the tibial nerve meets the skin surface; DP, curvilinear distance measured from MM to the point where the perpendicular line drawn from the middle of the deep peroneal nerve meets the skin surface; S, curvilinear distance from the MM to the point where the perpendicular line drawn from the middle of the saphenous nerve meets the skin surface; SR1, curvilinear distance from the LM to the point where the perpendicular line drawn from the middle of the sural nerve meets the skin surface; SP, curvilinear distance from the LM to the point where the perpendicular line drawn from the middle of the medial dorsal cutaneous nerve meets the skin surface; dT, perpendicular distance from the tibial nerve to the skin surface; dDP, perpendicular distance from the deep peroneal nerve to the skin surface; dS, perpendicular distance from the saphenous nerve to the skin surface; dSR, perpendicular distance from the sural nerve to the skin surface; dSP, perpendicular distance from the medial dorsal cutaneous nerve to the skin surface.

| Nerve | Curvilinear Distance | | | Depth | | |
| --- | --- | --- | --- | --- | --- | --- |
|  | Measurement | Mean (mm) | Standard deviation | Measurement | Mean (mm) | Standard deviation |
| Tibial | T1 | 32.5 | 8.9 | dT | 9.2 | 2.4 |
| Deep Peroneal | DP | 62.8 | 11.1 | dDP | 7.4 | 1.9 |
| Saphenous | S | 24.4 | 7.9 | dS | 2.8 | 1.1 |
| Sural | SR1 | 27.9 | 6.3 | dSR | 5.2 | 1.3 |
| Medial dorsal cutaneous | SP | 52.7 | 7.3 | dSP | 2.1 | 0.6 |
